# Advanced Nanomaterial-Based Biosensors for N-Terminal Pro-Brain Natriuretic Peptide Biomarker Detection: Progress and Future Challenges in Cardiovascular Disease Diagnostics

**DOI:** 10.3390/nano14020153

**Published:** 2024-01-10

**Authors:** Yen-Yi Lee, Balasubramanian Sriram, Sea-Fue Wang, Sakthivel Kogularasu, Guo-Ping Chang-Chien

**Affiliations:** 1 Institute of Environmental Toxin and Emerging-Contaminant Research, Cheng Shiu University, Kaohsiung 833301, Taiwan; 2 Super Micro Mass Research and Technology Center, Cheng Shiu University, Kaohsiung 833301, Taiwan; 3 Center for Environmental Toxin and Emerging-Contaminant Research, Cheng Shiu University, Kaohsiung 833301, Taiwan; 4 Department of Materials and Mineral Resources Engineering, National Taipei University of Technology, Taipei 10608, Taiwan

**Keywords:** nanomaterials, biomarkers, N-terminal pro-B-type natriuretic peptide, cardiovascular diseases, biosensors

## Abstract

Cardiovascular diseases (CVDs) represent a significant challenge in global health, demanding advancements in diagnostic modalities. This review delineates the progressive and restrictive facets of nanomaterial-based biosensors in the context of detecting N-terminal pro-B-type natriuretic peptide (NT-proBNP), an indispensable biomarker for CVD prognosis. It scrutinizes the escalation in diagnostic sensitivity and specificity attributable to the incorporation of novel nanomaterials such as graphene derivatives, quantum dots, and metallic nanoparticles, and how these enhancements contribute to reducing detection thresholds and augmenting diagnostic fidelity in heart failure (HF). Despite these technological strides, the review articulates pivotal challenges impeding the clinical translation of these biosensors, including the attainment of clinical-grade sensitivity, the substantial costs associated with synthesizing and functionalizing nanomaterials, and their pragmatic deployment across varied healthcare settings. The necessity for intensified research into the synthesis and functionalization of nanomaterials, strategies to economize production, and amelioration of biosensor durability and ease of use is accentuated. Regulatory hurdles in clinical integration are also contemplated. In summation, the review accentuates the transformative potential of nanomaterial-based biosensors in HF diagnostics and emphasizes critical avenues of research requisite to surmount current impediments and harness the full spectrum of these avant-garde diagnostic instruments.

## 1. Introduction

Cardiovascular diseases (CVDs) continue to be a predominant cause of morbidity and mortality worldwide, posing complex challenges in the realms of clinical diagnostics and healthcare management. According to recent reports by the World Health Organization (WHO), CVDs are the number one cause of death globally, accounting for an estimated 17.9 million lives each year [1,2,3]. This stark statistic underscores the urgent need for effective diagnostic and therapeutic strategies. The intricate pathophysiology of CVDs demands precise and early detection methods, thereby elevating the significance of biomarkers in cardiovascular healthcare. Biomarkers, as specific biological indicators, provide critical insights into an individual’s physiological and pathological states, playing an instrumental role in early disease detection, risk stratification, and therapeutic monitoring of cardiovascular conditions. Their relevance is not limited to reflecting underlying pathophysiological changes but also extends to informing and guiding clinical decision-making processes. The identification and utilization of these biomarkers are vital for improving patient outcomes, particularly in the early stages of cardiovascular disorders, where timely intervention can significantly alter disease progression and prognosis [4].

**Scheme 1:**
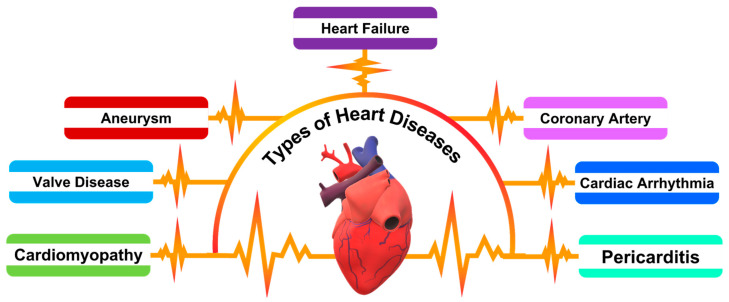
A comprehensive representation of common heart conditions contributing to heart failure, encompassing aneurysm, valve disease, cardiomyopathy, coronary artery disease, cardiac arrhythmia, and pericarditis.

As shown in Scheme 1, the spectrum of heart diseases associated with heart failure encompasses conditions such as aneurysms, which involve abnormal bulges in blood vessel walls; valve diseases, which disrupt blood flow through the heart; cardiomyopathies, diseases of the heart muscle that affect its pumping ability; coronary artery disease, the narrowing or blockage of coronary arteries leading to ischemia; cardiac arrhythmias, which can impair the heart’s pumping efficiency; and pericarditis, an inflammation of the lining around the heart. Each of these conditions can contribute to the complex clinical presentation of heart failure, underscoring the need for a diverse array of diagnostic biomarkers to accurately reflect the multifaceted nature of CVD pathophysiology.

NT-ProBNP, a prominent cardiac biomarker, has gained significant recognition for its role in the diagnosis and prognosis of HF. This biomarker is synthesized as a response to ventricular volume expansion and pressure overload, conditions often indicative of cardiac stress and dysfunction. NT-ProBNP, a cleavage byproduct of proBNP, is released primarily by cardiac myocytes. Its elevated levels in the bloodstream are not only diagnostic but also correlate proportionally with the severity of heart disease, offering a nuanced understanding of cardiac function. Distinguished by its superior specificity and sensitivity, NT-ProBNP is particularly valuable in diagnosing HF, especially in cases with unclear clinical manifestations. Beyond its diagnostic capabilities, NT-ProBNP serves as a crucial tool for patient risk stratification, guiding therapeutic decision-making, and monitoring the response to treatment in both acute and chronic HF scenarios. Its comprehensive utility in various clinical settings solidifies NT-ProBNP’s position as a vital biomarker in the landscape of cardiovascular healthcare and patient management [5,6,7]. A recent report by Hall et al. explores BNP, initially isolated from porcine brains and later identified as a cardiac hormone. The study explains that BNP and atrial natriuretic peptide together form a critical system in the heart. The synthesis and secretion of proBNP, mainly triggered by myocyte stretch, result in active BNP and NT-proBNP. The research highlights that heart failure leads to increased BNP secretion due to factors like wall stretch and neurohormonal activation. The paper also outlines BNP’s physiological effects, including diuresis and vasodilation, and details the clearance mechanisms for BNP and NT-proBNP, noting the latter’s longer half-life and higher plasma concentration [8].

A study by Rakesh et al. delves into the synergistic amalgamation of nanotechnology and biotechnology, culminating in advanced biosensing modalities for medical diagnostics. This work underscores the development and classification of diverse nanomaterials, emphasizing their distinct chemical and structural attributes, which are essential for detecting a wide array of biological phenomena, including pathogenic and toxic agents. The discourse extends to a critical analysis of biosensors, categorizing them based on bio-recognition elements and signal transduction mechanisms integral to medical applications. Additionally, it addresses the dynamic and evolving landscape of biosensing technology while scrutinizing the challenges encountered in the practical application of nanomaterials in medical biosensing frameworks [9].

The emergence of biosensor technology, characterized by the fusion of biological components with physicochemical detectors, represents a transformative advancement in the realm of biomedical diagnostics. These sophisticated devices are engineered to convert biological interactions into quantifiable electronic signals, thus facilitating rapid, sensitive, and specific detection of a diverse array of analytes. Among these, biomarkers such as NT-ProBNP, pivotal in cardiovascular disease diagnostics, are of particular interest. The incorporation of nanomaterials into biosensor frameworks has catalyzed a paradigm shift in biosensing capabilities [10]. Nanomaterials, endowed with distinct physicochemical attributes like an extensive surface area-to-volume ratio, augmented reactivity, and pronounced quantum mechanical effects, have significantly enhanced the analytical performance of biosensors [11,12,13,14,15]. They contribute to elevated sensitivity and selectivity, expanding the detection limits and improving the fidelity of biosensor readings [16,17,18,19,20].

This review presents a thorough analysis of the contemporary landscape and progressions in nanomaterial-based biosensors for NT-ProBNP detection. It delves into the evolutionary trajectory of these biosensors, examining their foundational mechanisms, relative effectiveness, and the scope of their clinical utility in managing cardiovascular diseases. Central to this exploration is the dissection of the inherent challenges and limitations faced by these biosensors, as well as an evaluation of the prospective advancements in this swiftly advancing domain. Additionally, the review highlights the transformative influence of nanomaterials in biosensing technologies, particularly in the context of cardiovascular health. This includes an assessment of how nanomaterials, through their unique properties, are reshaping the capabilities and applications of biosensors in detecting critical cardiac biomarkers like NT-ProBNP. The aim is to provide an insightful perspective on how these technological innovations are redefining diagnostic approaches and potentially altering the paradigm of cardiovascular disease management.

## 2. Cardiovascular Biomarkers: Molecular Insights and Diagnostic Implications

**Scheme 2:**
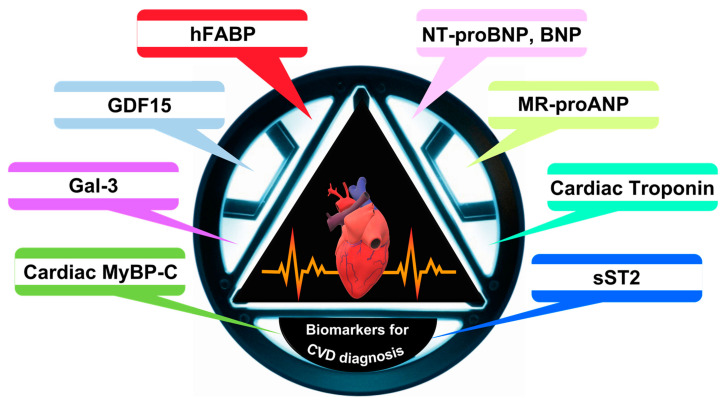
Overview of key biomarkers for CVD diagnosis. The typical biomarkers critical for CVD evaluation are: hFABP, GDF15, Gal-3, NT-proBNP, BNP, MR-proANP, cardiac troponin, sST2, and cardiac MyBP-C.

As illustrated in Scheme 2, within the realm of cardiovascular diagnostics, the significance of biomarkers, especially cardiac troponins (cTnI and cTnT) and natriuretic peptides (BNP and NT-ProBNP), is crucial. Cardiac troponins, which are integral regulatory proteins essential for cardiac muscle contraction, become exceedingly critical in the scenario of myocardial injury, notably in myocardial infarction (MI). In the event of myocardial damage, these proteins are liberated into the bloodstream, serving as highly specific indicators of cardiac muscle injury. This specificity is paramount in differentiating cardiac events from muscular damage in other types of muscle tissue.

The diagnostic importance of cardiac troponins is not solely in their detection but also in understanding the kinetics of their release and subsequent removal from the bloodstream. This provides critical insights into the timing and extent of myocardial injury. Consequently, elevated levels of cTnI and cTnT are now recognized as the gold standard in the diagnosis of myocardial infarction.

In conjunction with cardiac troponins, natriuretic peptides, namely BNP and NT-ProBNP, provide valuable data in the diagnosis and management of HF. These peptides are released in response to ventricular stress and volume overload, which are hallmark features of HF. They play a substantial role in the maintenance of cardiovascular homeostasis by facilitating natriuresis, diuresis, and vasodilation. High levels of BNP and NT-ProBNP are indicative of increased intracardiac pressure and ventricular dysfunction, thus serving as vital biomarkers for HF. NT-ProBNP, in particular, is renowned for its extended half-life and stability in the bloodstream, rendering it a dependable marker for the assessment of chronic heart failure [21,22,23,24,25]. The recent report by Biasucci examines emerging biomarkers in HF beyond the established BNP. It highlights the role of inflammation and noncoding RNAs in HF’s pathogenesis and their potential in diagnosis, prognosis, and personalized treatment. The work suggests that future HF management will likely embrace precision medicine, utilizing a broader array of biomarkers and advanced “omics” technologies to enhance patient care [26].

A recent article by John et al. highlights the pivotal role of biomarker detection in diagnosing CVD, which is a major health concern globally. This area of research is experiencing rapid advancements, with a focus on novel biomarkers that can be identified through state-of-the-art techniques like proteomics, biosensing, and microfluidics, in addition to traditional markers like troponin. Their work emphasizes the integration of these methods with advanced optical sensing technologies, including high-performance liquid chromatography and LASER/LED-induced fluorescence, as well as Raman spectroscopy. These optical methods, enhanced by nanotechnology and microfluidic technologies, are crucial for probing patterns of multiple markers in clinical samples such as whole blood and serum. The ability to detect a broader spectrum of biomarkers with high sensitivity and specificity is essential for the early and accurate diagnosis of CVD, ultimately aiding in effective therapy and improving patient prognosis [27].

In addition to these cardiac-specific biomarkers, C-reactive protein (CRP) and lipoprotein-associated phospholipase A2 (Lp-PLA2) have emerged as significant markers in the broader context of cardiovascular diseases. CRP, an acute-phase reactant, has gained prominence as a marker of systemic inflammation, which plays a crucial role in atherosclerosis and other cardiovascular pathologies. The association of elevated CRP levels with an increased risk of coronary events has been well established, underlining its role in cardiovascular risk assessment. The development of high-sensitivity CRP assays has further enabled the detection of subtle, yet clinically relevant, levels of inflammation, aiding in the risk stratification for cardiovascular events, even in patients without overt CVD. Lp-PLA2, an enzyme associated with low-density lipoprotein (LDL), participates in the inflammatory process within atherosclerotic plaques. The levels of Lp-PLA2 are linked to the risk of coronary heart disease and stroke, positioning it as a potential biomarker for atherosclerosis. Its role in the pathogenesis of atherosclerosis and the associated risk of cardiovascular events highlights the importance of Lp-PLA2 in the preventive and diagnostic strategies for CVD [28,29,30].

## 3. NT-ProBNP versus Other Biomarkers in Cardiovascular Diseases

NT-ProBNP, or N-terminal pro-b-type natriuretic peptide, is a clinically significant biomarker extensively utilized in the diagnosis and monitoring of cardiovascular diseases, particularly HF. This peptide is a fragment released during the cleavage of proBNP, a precursor molecule synthesized primarily in the cardiac ventricles in response to increased wall tension. Elevated ventricular pressure, as seen in various heart failure conditions, stimulates the production and release of proBNP, which is subsequently cleaved into BNP, an active hormone with natriuretic properties, and NT-ProBNP, an inert fragment. The measurement of NT-ProBNP in blood serves as a surrogate marker for ventricular strain and stress, correlating with the severity of cardiac dysfunction [31,32]. The diagnostic utility of NT-ProBNP is particularly pronounced in differentiating heart failure from other causes of dyspnea or cardiac-related symptoms. Its levels rise significantly in patients with HF, aiding clinicians in confirming or ruling out the diagnosis. Moreover, NT-ProBNP has been incorporated into various clinical guidelines and risk stratification algorithms due to its ability to provide prognostic information. Higher levels of NT-ProBNP are associated with increased mortality and hospitalization rates in HF patients, making it a valuable tool for predicting patient outcomes and guiding treatment strategies [33].

The standard detection of NT-ProBNP in clinical laboratories involves various immunoassay techniques. Enzyme-linked immunosorbent assays (ELISAs) are commonly used due to their high sensitivity and specificity. These assays utilize antibodies specifically targeted against NT-ProBNP, enabling the quantification of its concentration in patient blood samples. Chemiluminescent and electrochemiluminescence immunoassays are other methodologies employed, offering the benefits of enhanced sensitivity and faster processing times. However, these techniques present certain challenges. The interpretation of NT-ProBNP levels can be influenced by patient-specific factors such as age, sex, and especially renal function, as renal impairment can lead to elevated NT-ProBNP levels independent of heart function. This necessitates careful consideration and contextual understanding when evaluating test results [25,34].

**Figure 1:**
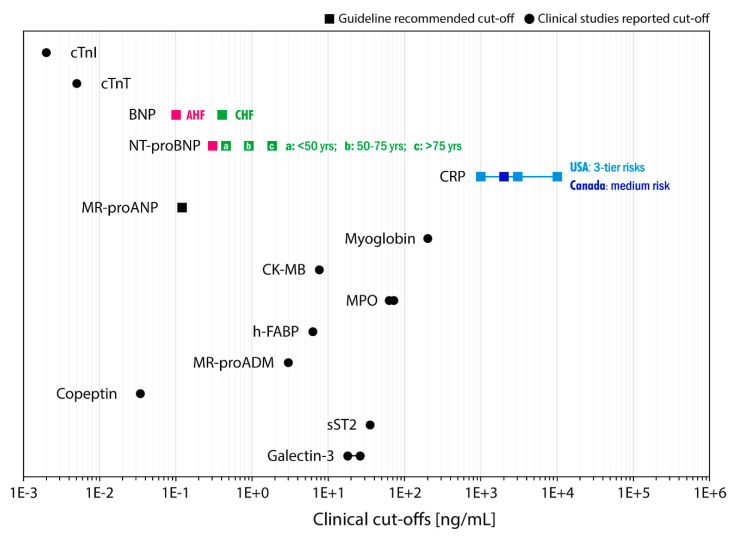
Established clinical thresholds for fourteen cardiac biomarkers reviewed here. This includes cardiac troponin I (cTnI), cardiac troponin T (cTnT), B-type natriuretic peptide (BNP), N-terminal propeptide B-type natriuretic peptide (NT-proBNP), mid-regional fragment of pro-atrial-natriuretic-peptide (MR-proANP), high-sensitivity C-reactive protein (hs-CRP), soluble suppressor of tumorigenicity 2 (sST2), galectin-3 (Gal-3), myoglobin (Myo), creatine kinase myocardial band (CK-MB), myeloperoxidase (MPO), heart-type fatty-acid-binding protein (h-FABP), and mid-regional fragment of pro-adrenomedullin-peptide (MR-proADM), in the context of acute heart failure (AHF) and chronic heart failure (CHF). The figure illustrates the age-dependent clinical thresholds (categorized as a–c), recommending 0.45, 0.9, and 1.8 ng/mL for age groups <50 years, 50–75 years, and >75 years, respectively. Reproduced with permission from the publisher [35].

The need for portable and affordable devices capable of measuring cardiac biomarkers is increasingly critical in the management of CVDs, particularly for underserved populations. Such devices are essential in addressing the substantial global burden of CVDs, including HF and acute coronary syndrome (ACS), which encompasses conditions like MI. Current medical guidelines from regions such as the United States, Canada, and Europe emphasize the role of both established and emerging cardiac biomarkers in the prognosis, diagnosis, and risk stratification of CVDs. As shown in Figure 1, among these, BNP and NT-proBNP are identified as key biomarkers for ACS, especially valuable for early risk stratification in suspected ACS cases. The European Society of Cardiology (ESC) also recognizes the significance of natriuretic peptides (NPs) in providing prognostic information alongside cardiac troponin for ACS [35]. Another challenge in NT-ProBNP detection is the variation in assay calibration and the lack of standardization across different testing platforms, potentially leading to inconsistencies in measurements between laboratories. This variability underscores the need for clinicians to be aware of the specific assay characteristics and reference ranges used in their practice settings.

## 4. Nanomaterial-Based Biosensors for NT-ProBNP Detection

**Figure 2:**
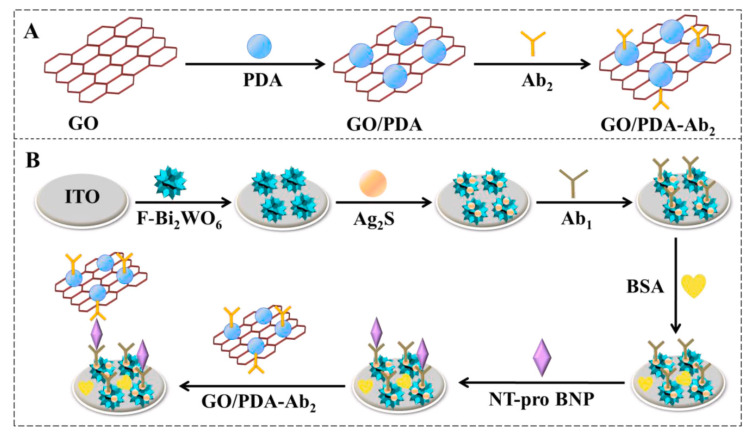
(**A**) Preparation procedure for GO/PDA-Ab2. (**B**) Construction steps for the sandwich-type PEC immunosensor tailored for NT-Pro BNP detection. Reproduced with permission from the publisher [36].

The detection of NT-ProBNP, a crucial biomarker for cardiovascular diseases, has been significantly enhanced by the integration of nanomaterials into biosensor designs. These advancements leverage the unique properties of nanomaterials to achieve high sensitivity and specificity in NT-ProBNP detection. In a recent article by Qian et al., a highly sensitive and selective immunosensor is designed for NT-pro BNP detection, employing flower-like Bi_2_WO_6_/Ag_2_S nanoparticles (F-Bi_2_WO_6_/Ag_2_S) as the photoelectrochemical matrix. This matrix is coupled with a graphene oxide and polydopamine (GO/PDA) composite for signal amplification, as shown in Figure 2. The sensor capitalizes on the efficient photocurrent conversion provided by the cascade-like band-edge level between F-Bi_2_WO_6_ and Ag_2_S, further enhanced by the GO/PDA conjugates. These conjugates aid in sweeping the holes and preventing the recombination of photogenerated electron-hole pairs, with graphene oxide’s excellent conductivity facilitating rapid electron transfer. The resulting photocurrent correlates directly with NT-pro BNP concentration, with the sensor demonstrating a detection range from 0.1 pg/mL to 100 ng/mL and a low detection limit of 0.03 pg/mL [36].

**Figure 3:**
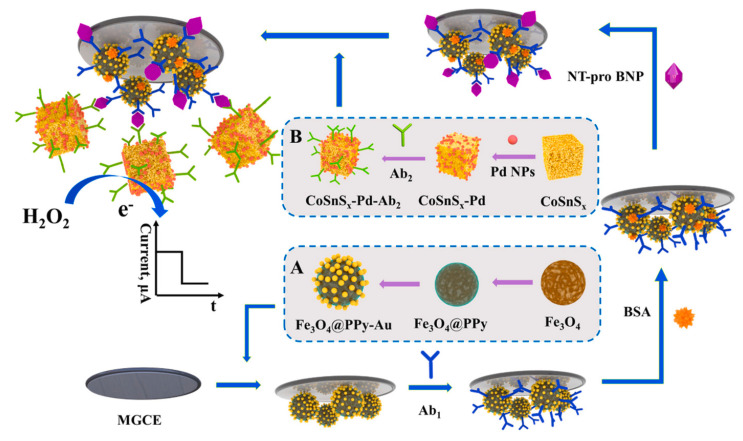
Illustrative depiction of the immunosensor assembly: the creation process of Fe_3_O_4_@PPy-Au (**A**) and CoSnS_x_-Pd-Ab_2_ (**B**). Reproduced with permission from the publisher [47].

In a study by Li et al., a novel electrochemical immunoassay is developed for NT-pro BNP detection, as shown in Figure 3. This sensor features an amorphous bimetallic sulfide of CoSnS_x_-Pd as a signal amplifier and Fe_3_O_4_@PPy-Au as a magnetic substrate. The CoSnSx, synthesized via a stoichiometric co-precipitation method, exhibits excellent electrochemical behavior due to the synergistic effect of SnS_2_ and cobalt, enhanced by Pd nanoparticles’ electrocatalytic activity towards H_2_O_2_ oxidation. The Fe_3_O_4_@PPy-Au substrate, immobilized on the magnetic glassy carbon electrode, enhances conductivity and stability. This design achieves a wide detection range from 0.1 pg/mL to 50 ng/mL and a low detection limit of 31.5 fg/mL for NT-pro BNP, showing potential for broad biomarker detection in medical diagnostics [37].

### 4.1. An Overview of Various Nanomaterial Types, Their Distinct Properties, and Applications in the Detection of NT-ProBNP

#### 4.1.1. Metal Nanoparticles

Gold (AuNPs) and silver (AgNPs) are renowned for their localized surface plasmon resonance (LSPR) properties, which are highly sensitive to changes in the refractive index near the nanoparticle surface. This sensitivity is leveraged in colorimetric assays where shifts in the LSPR spectrum serve as indicators of molecular interactions, such as the binding of NT-ProBNP. The colorimetric response resulting from these shifts can be easily observed, making AuNPs and AgNPs ideal for rapid and straightforward detection methods. The intensity and peak wavelength of the LSPR are influenced by factors including particle size, shape, and the dielectric environment, allowing for finely-tuned biosensor designs.

Platinum (Pt), palladium (Pd), and copper (Cu) nanoparticles offer distinct electrocatalytic properties and electrical conductance, which are advantageous in the development of electrochemical sensors for NT-ProBNP detection. Platinum nanoparticles, for instance, provide excellent catalytic activity towards various biochemical reactions, enhancing the electron transfer processes necessary for sensitive electrochemical detection. Palladium nanoparticles also exhibit similar catalytic properties, with a unique affinity for hydrogen absorption, which can be utilized in biosensing applications. Copper nanoparticles contribute to improved electrical conductance and have been employed in electrochemical sensors for their cost-effectiveness and efficient catalytic behavior. These metals can be utilized to fabricate highly sensitive and specific electrochemical biosensors for NT-ProBNP, capitalizing on their unique properties to detect minute changes in electrochemical signals that correspond to the presence of the biomarker [18,37].

#### 4.1.2. Carbon-Based Nanomaterials

Carbon nanotubes (CNTs) are recognized for their exceptional electrical conductivity, substantial surface area, and distinct electron transport characteristics. These properties make CNTs highly effective in electrochemical sensor applications. In the context of NT-ProBNP detection, CNTs are employed to enhance signal transduction. When NT-ProBNP antibodies are bound to the surface of CNTs, their unique electron transport properties facilitate efficient signal relay, amplifying the detection capabilities of the sensor. Additionally, the large surface area of CNTs offers ample sites for antibody attachment, thereby increasing the sensor’s sensitivity and allowing for the detection of low concentrations of NT-ProBNP.

Graphene and graphene oxide, with their high surface area and excellent electrical conductivity, are versatile materials in biosensor technology. Their planar structure allows for substantial functionalization with various biomolecules, including antibodies specific to NT-ProBNP. This functionalization is crucial for creating selective and sensitive biosensing interfaces. Graphene-based materials are particularly useful in field-effect transistor (FET)-based sensors, where the conductive properties of graphene can be modulated by the binding of target biomolecules, leading to measurable changes in electrical signals. Additionally, their use in fluorescence-quenching sensors for NT-ProBNP detection is noteworthy. In these applications, graphene or graphene oxide can quench the fluorescence of labeled antibodies or NT-ProBNP, with the quenching effect being reversed upon the binding of the target molecule. This property is exploited to create highly sensitive sensors that can detect minute changes in fluorescence, correlating with the concentration of NT-ProBNP in a sample [20,38].

#### 4.1.3. Semiconductor Nanoparticles

Quantum Quantum Dots (QDs) are semiconductor nanoparticles renowned for their quantum confinement effects, which allow for size-tunable fluorescence emission, providing a unique advantage in biosensor design. This size dependency of their optical properties enables precise control over the emission wavelength, making QDs highly customizable for various applications. Their high photostability further adds to their appeal, ensuring consistent fluorescence over extended periods. In NT-ProBNP detection, QDs are employed as fluorescent probes within biosensors. These biosensors leverage changes in the fluorescence properties of the QDs, induced by the binding of NT-ProBNP to the sensor, to signal the presence of this biomarker. The sensitivity of QDs to even minor changes in fluorescence makes them particularly effective in detecting low concentrations of NT-ProBNP, offering a powerful tool for early and accurate heart failure diagnosis [19].

#### 4.1.4. Magnetic Nanoparticles

Magnetic nanoparticles, particularly those based on iron oxide, are crucial in biosensing due to their superparamagnetic properties. These properties allow for easy manipulation and concentration of the nanoparticles using external magnetic fields, a feature immensely beneficial in assays for NT-ProBNP detection. In such applications, these nanoparticles are functionalized with specific biomolecules that bind to NT-ProBNP. Once bound, they can be magnetically concentrated, significantly enhancing the precision and sensitivity of the assay. This concentration is particularly advantageous in subsequent steps of electrochemical or optical detection, where the gathered nanoparticles facilitate amplified signals or observable changes in optical properties. This method not only improves the detection sensitivity but also allows for more efficient and accurate quantification of NT-ProBNP levels, making iron oxide-based magnetic nanoparticles a vital component in advanced biosensor systems for heart failure diagnostics [39].

#### 4.1.5. Silicon Nanoparticles

Silicon nanoparticles stand out in biosensing applications due to their exceptional photoluminescent properties combined with notable biocompatibility, making them ideal candidates for bioconjugation with molecules for fluorescence-based detection. Their quantum confinement-induced luminescence is particularly advantageous for optical biosensing techniques. When used in NT-ProBNP detection, these nanoparticles can be conjugated with specific biomolecules, such as antibodies or aptamers, targeting NT-ProBNP. Upon binding with NT-ProBNP in a sample, these nanoparticles exhibit changes in fluorescence intensity or energy transfer, providing a quantifiable signal. This change in fluorescence is directly correlated with the concentration of NT-ProBNP, enabling the precise and sensitive detection of this biomarker. The biocompatibility of silicon nanoparticles further enhances their suitability for use in complex biological environments, making them a valuable tool in the development of advanced optical biosensors for heart failure diagnostics [40].

#### 4.1.6. Lipid-Based Nanomaterials

Lipid-based nanomaterials, particularly liposomes and lipid bilayers, are increasingly utilized in biosensor technology due to their ability to mimic biological membranes, offering a biocompatible interface that closely resembles the natural cellular environment. This unique characteristic makes them especially suitable for biosensors that require membrane-like structures or are designed to interact directly with biological systems. Liposomes, which are spherical vesicles composed of lipid bilayers, can encapsulate various substances, including enzymes, drugs, or signaling molecules, making them versatile carriers for targeted delivery in biosensing applications. In the context of NT-ProBNP detection, these lipid-based nanomaterials can provide a conducive environment for the biomarker, facilitating more natural interactions and binding events. The integration of liposomes and lipid bilayers in biosensors enhances the bio-relevance of the sensing environment, thereby improving the sensor’s sensitivity and specificity towards NT-ProBNP. Their compatibility with a wide range of detection methods, including optical and electrochemical techniques, further underscores their utility in developing advanced biosensors for heart failure diagnostics [41].

#### 4.1.7. Polymeric Nanoparticles

Polymeric nanoparticles, crafted from biocompatible polymers, offer significant versatility and functionality in the realm of biosensing. Their composition allows for customization in terms of encapsulation and controlled release properties, making them highly adaptable for various diagnostic applications. In the detection of NT-ProBNP, a critical biomarker for HF, these nanoparticles can be specifically engineered to carry and protect biomolecules or drugs that are relevant to the sensing mechanism. This capability not only ensures the stability and bioavailability of these agents but also enhances the specificity and sensitivity of the sensors. By encapsulating recognition elements or enzymes that react with NT-ProBNP, polymeric nanoparticles can provide a focused and efficient means of detecting the biomarker. Furthermore, their ability to release these agents in a controlled manner can lead to more consistent and reliable sensor responses. This controlled release mechanism also opens possibilities for multiphasic sensing strategies, where different stages of detection can be orchestrated for improved accuracy. The inherent biocompatibility and tailorability of polymeric nanoparticles make them invaluable tools in the design of advanced biosensors, particularly for applications requiring high specificity and sensitivity, such as the detection of NT-ProBNP in heart failure diagnostics [42].

#### 4.1.8. Nanowires and Nanorods

Nanowires and nanorods, particularly those made of metals like gold or silver, are highly valued in biosensor technology due to their high aspect ratio, which confers an extensive surface area relative to their volume. This extensive surface area is crucial for the immobilization of biomolecules, such as antibodies or aptamers specific to NT-ProBNP, facilitating a higher density of functionalization. This property significantly enhances the sensitivity and efficiency of biosensors, as it allows for more interactions between the target biomarker and the sensor’s surface. Additionally, the conductive properties of metallic nanowires and nanorods are advantageous in signal transduction, particularly in electrochemical and optical biosensors. In electrochemical sensors, their conductive nature can lead to improved electron transfer, enhancing the signal-to-noise ratio. In optical biosensors, these nanostructures can interact with light through mechanisms like surface plasmon resonance, providing a basis for sensitive detection methods based on changes in optical properties. The incorporation of nanowires and nanorods into biosensor designs, therefore, offers a means to significantly amplify the detection capabilities of NT-ProBNP, contributing to more accurate and sensitive diagnostics for HF [43].

#### 4.1.9. Nanodiamonds

Nanodiamonds, emerging as a novel class of nanomaterials in the biosensing field, are distinguished by their stable photoluminescent properties and high biocompatibility, making them well-suited for medical and biological applications. These unique characteristics stem from their inert carbon-based structure and surface defects, which can be engineered to emit stable fluorescence. This fluorescence stability is a significant advantage in biosensing as it ensures consistent signal quality over time, which is essential for reliable diagnostics. Nanodiamonds can be functionalized with specific biomolecules, such as antibodies or aptamers targeting NT-ProBNP, to facilitate selective biomolecular interactions. In fluorescence-based detection systems for NT-ProBNP, the binding events between these functionalized nanodiamonds and the target biomarker result in modulation of their fluorescence properties. This change in fluorescence, either in intensity, wavelength, or lifetime, provides a quantifiable means of detecting NT-ProBNP presence and concentration. The ability to tailor the surface chemistry of nanodiamonds for specific interactions, combined with their photostability and biocompatibility, positions them as a promising material in the development of advanced, fluorescence-based biosensors for accurate and sensitive detection of HF biomarkers like NT-ProBNP [44].

#### 4.1.10. Plasmonic Nanostructures

Plasmonic nanostructures, such as nanostars or nanocages, represent an advanced class of materials in the field of optical biosensing, primarily due to their enhanced localized surface plasmon resonance (LSPR) effects. These complex nanostructures, with their unique geometries, offer a greater surface area and more hotspots for plasmonic activity compared to simpler nanoparticles. The intricate shapes of nanostars or the hollow interiors of nanocages lead to more pronounced and tunable LSPR properties, making them highly effective for specific optical sensing applications. In the context of NT-ProBNP detection, these plasmonic nanostructures are utilized to capitalize on their sensitive optical response to changes in the local refractive index, which occurs upon biomolecular binding events. When functionalized with recognition elements for NT-ProBNP, the interaction with the biomarker induces shifts in the LSPR spectrum, which can be detected as changes in absorption, scattering, or fluorescence. This sensitivity to minute refractive index changes allows for the detection of low concentrations of NT-ProBNP with high precision. The use of these plasmonic nanostructures in optical biosensors therefore offers a path to highly sensitive and specific NT-ProBNP detection, crucial for the early and accurate diagnosis of heart failure [45].

#### 4.1.11. Hybrid Nanocomposites

Hybrid nanocomposites, formed by integrating different nanomaterials, capitalize on the synergistic properties emerging from their combination, leading to enhanced performance in biosensing applications. By merging materials like magnetic nanoparticles, conductive polymers, or quantum dots, these composites achieve functionalities that surpass those of their individual components. For instance, the catalytic activity can be amplified, the electrical conductivity can be optimized, and the optical characteristics can be finely tuned. In NT-ProBNP detection, these hybrid nanocomposites are particularly valuable for implementing multifaceted detection strategies. They can combine magnetic properties for the concentration and separation of target biomarkers with optical properties for signal detection, such as fluorescence or plasmonic effects. This dual functionality enables more efficient capture and sensitive detection of NT-ProBNP, enhancing both the specificity and sensitivity of the assay. The versatility of hybrid nanocomposites allows for the development of advanced biosensing platforms that can effectively integrate various detection mechanisms, offering a powerful approach for comprehensive and accurate NT-ProBNP analysis in heart failure diagnostics [46].

**Figure 4:**
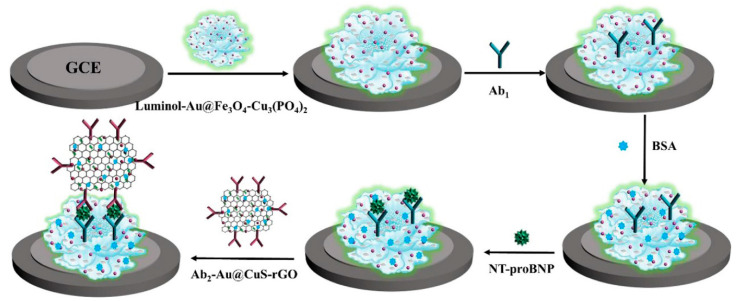
Electrochemiluminescence quenching of luminol by CuS in situ grown on reduced graphene oxide for detection of NT-proBNP. Reproduced with permission from the publisher [48].

As shown in Figure 4, in the study by Li et al., a new electrochemiluminescence (ECL) strategy for NT-proBNP detection is introduced, utilizing CuS grown on reduced graphene oxide (CuS-rGO) to quench a luminol/H_2_O_2_ system. The system involves luminol grafted onto Au@Fe_3_O_4_-Cu_3_(PO_4_)_2_ nanoflowers, enhancing ECL intensity through the catalytic reduction of H_2_O_2_. The quenching mechanism by CuS-rGO is based on ECL resonance energy transfer (RET), confirmed by spectral overlaps. The sensor shows a remarkable decrease in ECL signal post-immunoreaction and exhibits a broad detection range from 0.5 pg/mL to 20 ng/mL, with a low detection limit of 0.12 pg/mL. This approach combines high sensitivity, stability, and specificity, marking a significant advancement in NT-proBNP biosensing [48].

## 5. Technological Advancements in Nanomaterial-Based Biosensors for NT-ProBNP Detection

Recent advancements in nanomaterial-based biosensors have significantly enhanced the detection and monitoring of NT-ProBNP, a critical biomarker for heart failure and other cardiovascular diseases. One notable development is the creation of hybrid nanocomposites, which combine the distinct properties of different nanomaterials like metallic nanoparticles and carbon-based structures. These composites exhibit superior sensitivity and specificity due to synergistic effects in electron transfer and surface reactivity, crucial for accurately detecting minute concentrations of NT-ProBNP [44,49,50]. Additionally, the integration of nanomaterial-based sensors with microfluidic technology has led to the emergence of compact lab-on-a-chip devices. These devices enable the simultaneous processing and analysis of small-volume blood samples, streamlining the detection process and providing rapid, on-site diagnostic results. The incorporation of wireless communication in these sensors has further facilitated remote monitoring of NT-ProBNP levels, crucial for the continuous management of chronic heart conditions [32,51,52].

**Figure 5:**
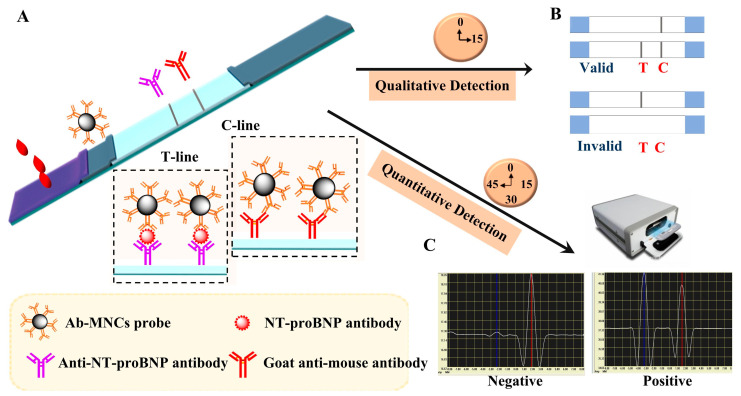
Analysis of the PAA-MNCs-based lateral flow immunoassay. (**A**) Illustrative representation of the lateral flow immunoassay targeting amine-terminal pro-B-type natriuretic peptide. (**B**) Qualitative assessment of the LFIAs. (**C**) Image of the corresponding MAR assay setup. Reproduced with permission from the publisher [53].

In a recent study, Yang et al. developed an improved lateral flow immunoassay (LFIA) using poly (acrylic acid)-stabilized superparamagnetic Fe_3_O_4_ nanocrystal clusters (PAA-MNCs). This novel LFIA addresses the limitations of traditional gold nanoparticle-based assays by offering both qualitative and quantitative detection capabilities, as shown in Figure 5. The PAA-MNCs, synthesized through a one-pot process, exhibit enhanced stability and dispersity, making them suitable for sensitive biomarker detection. Applied to NT-proBNP antibody detection, the LFIA shows a qualitative detection limit of 100 pg/mL and a quantitative detection range from 20 to 8000 pg/mL. This advancement in LFIA technology is particularly promising for clinical assessment of heart failure and personalized medicine [53].

Emerging trends in the design and functionality of these biosensors also reflect a growing emphasis on versatility and user accessibility. Recent innovations include the development of multiplexed biosensors capable of simultaneously detecting NT-ProBNP alongside other relevant cardiac biomarkers. This multi-analyte approach is invaluable in comprehensive cardiovascular diagnostics, where a holistic view of various biomarkers can provide a more accurate assessment of cardiac health. Furthermore, the coupling of nanomaterial-based biosensors with smartphone technology is revolutionizing point-of-care testing. By enabling the analysis and interpretation of NT-ProBNP levels through user-friendly mobile applications, these advancements are making cardiovascular monitoring more accessible outside traditional clinical settings. Additionally, the application of artificial intelligence and machine learning algorithms in interpreting biosensor data is enhancing the predictive accuracy of NT-ProBNP measurements, offering potential for personalized treatment regimens and improved patient outcomes in cardiovascular care [17,54,55]. A comparative analysis of various nanomaterial-based biosensors for NT-ProBNP detection is given in Table 1.

**Figure 6:**
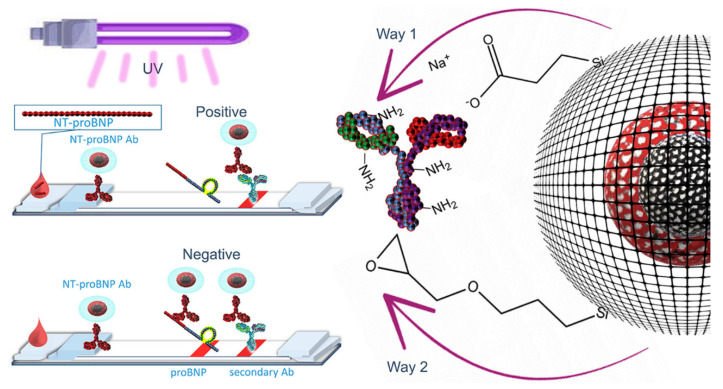
Express test for NT-proBNP competitive detection based on lateral flow immunoassay using silanized fluorescent quantum dots. Reproduced under a creative commons license [56].

In the study by Olga et al., an advanced LFIA is developed for detecting NT-proBNP, a crucial biomarker for heart failure. The assay utilizes CdSe/CdS fluorescent QDs coated with silicon dioxide, achieving a high fluorescence quantum yield of 51%, as shown in Figure 6. These QDs are functionalized and conjugated with antibodies specific to NT-proBNP. The LFIA operates on competitive detection, with optimized components to reduce nonspecific interactions. Applied to human plasma with a 10-fold dilution, the test has a cut-off level of 0.5 ng/mL NT-proBNP, where the disappearance of test line fluorescence indicates a positive result. This development offers a sensitive and efficient method for NT-proBNP detection in clinical settings [56].

**Table 1:** Comparative analysis of various nanomaterial-based biosensors for NT-ProBNP detection.

Nanomaterials	Analyte	Methods	Medium	Biorecognition Element	Linear Range	LOD	Refs.
AuNCs-HRP-Ab2	NT-proBNP	Voltametry	PBS (hydroquinone)	Antibody	0.02 to 100 ng/mL	6 pg/mL	[42]
AFMN/Bio-Ab1/Ag/PPN-Ab2	NT-proBNP	Amperometry	PBS	Antibody	0.005–4 ng/mL	0.003 ng/mL	[57]
AlGaN/GaN HEMT	NT-proBNP	FET	PBS 1 × (4% BSA) Serum	Aptamer	0.25–10 ng/mL	0.25 ng/mL	[58]
CoFe_2_O_4_@AuNPs	NT-proBNP	SERS	PBS pH 7.4	Antibody	1 fg/mL^–1^ ng/mL	0.75 fg mL^–1^	[59]
poly-Si NW	NT-proBNP	Voltametry	PBS pH 7.4	Antibody	32 pM–32 nM	32 pM	[60]
Fe_3_O_4_@PPy-Au and CoSnS_x_-Pd-Ab_2_	NT-proBNP	Amperometry	PBS pH 7.4	Antibody	0.1 pg/mL–50 ng/mL	31.5 fg/mL	[47]
rGO thin-films	NT-proBNP	FET	Serum	Antibody	10–1000 pg/mL	1 pg/mL	[61]
Graphene	NT-proBNP	FET	0.1 × PBS	Aptamer	0.1–104 pg/mL	0.01 pg/mL	[38]
AuPd NCs/NPC	NT-proBNP	EIS	PBS pH 7.4 containing 10.0 mmol/L H_2_O_2_ Serum	Antibody	0.001–10 ng/mL	0.34 pg/mL	[20]
Si_3_N_4_/SiO_2_	NT-proBNP	FET	Saliva	Antibody	0.02–1 pg/mL 0.5–20 pg/mL	0.02 pg/mL	[32]
SPGE	NT-proBNP	EIS	PBS	Antibody	0.1–5 ng/mL	0.1 ng/mL	[62]
SiO_2_@Ir	NT-proBNP	ECL	PBS (0.1 M)	Antibody	0.1–200 ng/mL	0.03 ng/mL	[63]
PdCu@SWCNH	NT-proBNP	ECL	PBS pH 8.0 (0.1 M + 2.0 mM H_2_O_2_)	Antibody	0.1 pg/mL–25 ng/mL	0.05 pg/mL	[46]
Ti:BiOBr-Au	NT-proBNP	ECL	PBS pH 7.0 (100 mM KCl + 80 mM K_2_S_2_O_8_)	Antibody	0.001–50 ng/mL	0.33 pg mL	[37]
TiO_2_@CN-Au	NT-proBNP	ECL	PBS pH 7.4 (0.1 M + 0.1 M K_2_S_2_O_8_) Serum	Antibody	0.0001–10 ng/mL	50 fg/mL	[64]
SnO_2_/SnS_2_/Mpg-C_3_N_4_ amplified by PbS/SiO_2_	NT-proBNP	PEC	PBS pH 7.4 (AA 0.2M)	Antibody	0.1 pg/mL–50 ng/mL	0.05 pg/mL	[65]
F-Bi_2_WO_6_/Ag_2_S and GO/PDA	NT-proBNP	PEC	PBS pH 7.4 (0.1 M)	Antibody	0.1 pg/mL–100 ng/mL	0.03 pg/mL	[36]
La-CdS/3D ZnIn_2_S_4_/Au@ZnO	NT-proBNP	PEC	PBS pH 7.4 (0.1 M + AA) Serum 500X	Antibody	0.8 pg/mL–45 ng/mL	0.32 g/mL	[66]
SiO_2_	NT-proBNP	SPR	DI Water	Antibody	0–10 ng/mL	0.1275 ng/mL	[40]
MNPs	NT-proBNP	Resistance	Plasma	Antibody	5–40,000 pg/mL	5 pg/mL	[40]

Abbreviations: gold nanochains (AuNCs) and horseradish peroxidase (HRP) complex labeled secondary antibodies; phosphate-buffered saline (PBS); Avidin functional magnetic nanoparticles (AFMN)/labeled detection antibodies (PPN-Ab2) and biotinylated capture antibodies (Bio-Ab1)/platinum and prussian blue nanomaterial (PPN); aluminum gallium nitride (GaN)/high electron mobility transistors (HEMTs); field effect transistor (FET); bovine serum albumin (BSA); surface-enhanced Raman spectroscopy (SERS); polysilicon nanowire (poly-Si NW); reduced graphene oxide (rGO) thin-films; AuPd nanocrystals/N-doped honeycombed porous carbon; electrochemical impedance spectroscopy (EIS); screen-printed gold electrodes (SPGE); electrochemiluminescent (ECL); PdCu nanocubes supported single-walled carbon nanohorns (PdCu@SWCNHs); gold nanopariticles (AuNPs)/Ti-doped bismuth oxybromide (BiOBr) (Ti:BiOBr-Au); carbon nitride-gold (CN-Au); photoelectrochemical (PEC); graphene oxide and polydopamine (GO/PDA); surface plasmon resonance (SPR); magnetic nanoparticles (MNPs).

## 6. Challenges in Development and Application for NT-ProBNP Detection

The development of nanomaterial-based biosensors for NT-ProBNP, a key biomarker in heart failure diagnosis and management, faces several scientific and technical hurdles. Among these, reproducibility in the synthesis of nanomaterials stands out as a primary challenge. Achieving consistent quality in nanomaterial production is vital, as the sensitivity and specificity of these biosensors are significantly dependent on factors like particle size, shape, and surface chemistry. Variability in these parameters can lead to fluctuations in biosensor performance, making reproducibility a crucial aspect of research and development.

Reproducibility issues often stem from the complex nature of nanomaterial synthesis, where slight variations in synthesis conditions can result in significant differences in material properties. Ensuring uniformity across batches requires rigorous standardization of synthesis protocols and precise control of experimental conditions. This level of consistency is essential for reliable biosensor performance, particularly for clinical applications where accuracy is paramount. In addition to the challenges in nanomaterial synthesis, the biofunctionalization process, crucial for selective NT-ProBNP recognition, demands precise control. This involves attaching biological recognition elements, like antibodies or aptamers, to the nanomaterials. It is imperative to maintain the biological activity and binding affinity of these molecules during conjugation to ensure the biosensor’s selectivity and sensitivity towards NT-ProBNP.

Moreover, the integration of these biosensors into practical, robust diagnostic tools presents challenges. The biosensors must perform reliably in complex biological matrices like blood or serum, where NT-ProBNP is measured. Overcoming issues related to sample interference and sensor stability in these matrices is key for successful application. Thus, the development of NT-ProBNP biosensors requires a multidisciplinary approach, combining material science, biochemistry, and engineering expertise to effectively address the challenges of reproducibility, biofunctionalization, and sensor integration. The overview of biosensor types for NT-ProBNP detection, including detection principles, advantages, and disadvantages, is given in Table 2.

### Limitations in Sensitivity, Cost, and Real-World Application

In the field of heart failure diagnostics, nanomaterial-based biosensors for NT-proBNP detection, despite their innovative contributions, encounter several scientific and practical challenges that need addressing for wider clinical integration. Sensitivity remains a critical limitation, and achieving the necessary detection thresholds for clinical use is challenging. Factors like the signal-to-noise ratio, nonspecific binding, and precision in nanomaterial properties often impede the ability to detect extremely low concentrations of NT-proBNP. This necessitates further advancements in nanomaterial synthesis and functionalization techniques to enhance detection limits and specificity under varied clinical conditions. Cost-effectiveness is another significant hurdle, as the sophisticated processes involved in the production and biofunctionalization of nanomaterials escalate the overall cost of biosensor development. This economic factor becomes particularly crucial when considering the deployment of these technologies in resource-constrained environments, highlighting the need for more cost-effective manufacturing methods. Additionally, the practical application of these biosensors in real-world settings presents challenges in device robustness, user friendliness, and regulatory compliance. Ensuring the biosensors’ functionality in diverse point-of-care environments, often operated by non-specialists, and navigating the regulatory pathways for clinical approval are essential steps for their adoption in healthcare systems. Thus, while nanomaterial-based biosensors for NT-proBNP detection hold transformative potential for cardiovascular disease management, overcoming these sensitivity, cost, and practical applicability challenges is vital for their successful implementation in routine clinical practice and broader healthcare settings.

## 7. Future Developments in Nanomaterial-Based Biosensors

The trajectory of cardiovascular diagnostics is poised for a paradigm shift with the advancement of nanomaterial-based biosensors, especially for the detection of NT-proBNP, a biomarker critical in heart failure management. Foreseen developments are anchored in the exploitation of novel nanomaterials, such as graphene derivatives, quantum dots, and customized metallic nanoparticles, each selected for their unique physicochemical properties that enhance sensor sensitivity and specificity. These nanomaterials, characterized by their exceptional electrical conductance and optical properties, are anticipated to facilitate the ultra-sensitive detection of NT-proBNP, even at minute concentrations. A significant development trajectory is the amalgamation of these biosensors with wearable technology. This integration aims to establish continuous, non-invasive monitoring systems for NT-proBNP, providing instantaneous cardiac status data crucial for prompt clinical decision-making. Concurrently, there is a growing focus on incorporating advanced computational methodologies, notably artificial intelligence and machine learning algorithms. These techniques are expected to revolutionize biosensor data analysis, enhance diagnostic precision, and potentially unveil novel biomarkers indicative of heart failure pathology.

Further, the optimization of nanomaterial biocompatibility and stability is crucial to ensuring their efficacy in long-duration monitoring scenarios. The drive towards creating cost-effective, user-friendly biosensors for point-of-care applications is integral, particularly to extend advanced cardiac diagnostic capabilities to under-resourced settings, thereby addressing global health disparities. In summation, these innovations in nanomaterial-based biosensors are set to expand their functional spectrum dramatically, establishing them as quintessential tools for personalized, proactive cardiovascular healthcare. This evolution represents a crucial step in transitioning towards more sophisticated, precise, and accessible diagnostics in cardiovascular medicine.

### Interdisciplinary Approaches and Integration with Other Technologies

The progression of nanomaterial-based biosensors for NT-proBNP detection in heart failure management epitomizes a multidisciplinary fusion, amalgamating advancements from various scientific fields and technologies. This integration is pivotal for enhancing the diagnostic capabilities of these biosensors. At the core of this evolution is the intersection of nanotechnology with bioengineering and materials science. Bioengineers focus on developing biosensors that not only exhibit heightened sensitivity and specificity but also maintain biocompatibility for prolonged clinical use. Concurrently, materials scientists are pivotal in innovating nanomaterials that possess enhanced electrical and optical properties, thereby elevating the performance standards of these biosensors.

Incorporating data science and artificial intelligence represents a significant leap in biosensor technology. The utilization of machine learning algorithms for the analysis of complex biosensor data enables the extraction of more nuanced diagnostic information. This approach allows for the refinement of diagnostics and paves the way for personalized patient care strategies based on intricate data patterns. Microfluidics technology, another critical component, contributes to the miniaturization of biosensors. This technology enables the development of compact, point-of-care devices capable of efficiently analyzing minuscule sample volumes, thereby facilitating rapid and on-site diagnostic testing.

Additionally, the integration of biosensor technology with wireless communication systems is instrumental in creating wearable biosensors. These devices allow for the continuous and non-invasive monitoring of NT-proBNP levels, providing real-time data transmission to healthcare providers. Such advancements enable prompt clinical interventions and ongoing patient monitoring, essential in the management of heart failure. Furthermore, the combination of biosensor data with advanced imaging techniques, such as magnetic resonance imaging (MRI) or positron emission tomography (PET), offers a more holistic diagnostic approach. By correlating biomarker levels with detailed cardiac imaging, a comprehensive understanding of the patient’s cardiac health can be achieved. This integration not only enhances diagnostic accuracy but also aids in tailoring patient-specific therapeutic interventions.

## 8. Conclusions

In conclusion, the biomarker NT-proBNP is critically important in the clinical landscape for diagnosing and managing heart failure, primarily due to its sensitivity in detecting and differentiating various cardiac dysfunctions. The advent of nanomaterial-based biosensors has markedly impacted the field of cardiovascular diagnostics. These biosensors, leveraging the unique properties of nanomaterials, offer superior sensitivity and specificity in NT-proBNP detection, enabling the identification of minute concentrations of the biomarker. This heightened sensitivity is crucial for early detection, which is key in preemptive heart failure management and in mitigating the risk of adverse outcomes. Additionally, the integration of nanotechnology in biosensor development has paved the way for the miniaturization of devices, leading to the creation of wearable and point-of-care biosensors. These advancements facilitate continuous, real-time monitoring of NT-proBNP levels, a significant step towards personalized medicine. This capability allows for dynamic adjustments in treatment strategies tailored to the patient’s fluctuating biomarker levels. As research in this domain progresses, incorporating interdisciplinary approaches and advanced technological integrations, nanomaterial-based biosensors are poised to further revolutionize heart failure diagnostics, potentially leading to substantial improvements in patient prognosis and quality of life in cardiovascular care.

## Figures and Tables

**Table 2:** Overview of biosensor types for NT-ProBNP detection: detection principles, advantages, and disadvantages.

Biosensor Type	Principle of Detection	Advantages	Disadvantages
Impedimetric Sensors [62]	Monitors alterations in electrical impedance as a result of antigen-antibody interaction specific to NT-proBNP.	Enables label-free quantification with heightened sensitivity due to direct electrical signal transduction.	May suffer from non-specific adsorption issues leading to false signals in complex biological samples.
Aptamer-Based Sensors (Apta-Sensors) [67]	Utilizes oligonucleotide sequences with high specificity for NT-proBNP to facilitate biomarker binding.	Offers molecular-level specificity and reduced nonspecific signal due to precise aptamer-biomarker affinity.	Aptamer design and synthesis can be complex and costly, and stability may be an issue in varied conditions.
Immunosensors [20]	Deploys antibody-antigen recognition for NT-proBNP, converting binding events into measurable signals.	Ensures high selectivity amidst biological sample complexity through targeted antibody-antigen interactions.	Production of specific antibodies can be expensive, and immunosensors may have limited reusability.
Potentiometric Sensors [68]	Measures the variance in electrochemical potential across electrodes induced by NT-proBNP binding activity.	Provides rapid electrochemical responses with miniaturization potential for point-of-care applications.	Potentiometric sensors can be affected by changes in pH or ion concentration, potentially affecting accuracy.
Electrochemiluminescence Sensors [39,69]	Detects NT-proBNP through emitted light from electrochemically activated luminescent reactions.	Enables sensitive and quantitative analysis of low-abundance biomarkers through luminescent signal amplification.	Requires sophisticated instrumentation and may have issues with background luminescence in complex samples.
Fiber-Optic Sensors [17,70]	Transduces NT-proBNP binding into modulated light signals within optical fibers.	Allows for continuous, non-invasive monitoring through optical signal variations in real-time detection.	Sensitivity may be limited by optical losses, and fiber-optic sensor complexity can increase costs.
Colorimetric Sensors [71]	Visual detection of NT-proBNP via chromogenic reactions leads to a colorimetric change.	Provides a straightforward interpretive approach and is cost-effective for rapid and widespread screening.	Lower sensitivity compared to other methods; quantification may be less accurate and subject to interpretation.
Surface Plasmon Resonance (SPR) Sensors [45]	Measures refractive index changes due to NT-proBNP interaction at a metal-dielectric interface.	Facilitates real-time, label-free kinetic analysis of biomarker interactions with high-throughput capability.	Requires expensive and sophisticated equipment; surface fouling can lead to decreased sensitivity over time.
Lateral Flow Sensors [56,72]	Uses capillary action to transport NT-proBNP to a detection zone, resulting in a visual signal.	Ideal for point-of-care settings, offering simplicity and rapid qualitative results without complex equipment.	Limited quantitative capacity; sensitivity may be lower than lab-based assays.
Microfluidic Sensors [57,67]	Integrates microscale fluid manipulation for NT-proBNP detection, often in a multiplexed format.	Enhances assay throughput and analytical precision by controlling reactant mixing and transport at microscale levels.	Fabrication of microfluidic devices can be complex and may require specialized equipment and processes.

## References

[1] Nabel E.G. (2003). Cardiovascular disease. N. Engl. J. Med..

[2] McGarrah R.W., White P.J. (2023). Branched-chain amino acids in cardiovascular disease. Nat. Rev. Cardiol..

[3] Consortium G.C.R. (2023). Global effect of modifiable risk factors on cardiovascular disease and mortality. N. Engl. J. Med..

[4] Wei C., Jiang Z., Li C., Li P., Fu Q. (2023). Nanomaterials responsive to endogenous biomarkers for cardiovascular disease theranostics. Adv. Funct. Mater..

[5] Richards M., Troughton R.W. (2004). NT-proBNP in heart failure: Therapy decisions and monitoring. Eur. J. Heart Fail..

[6] Pfister R., Scholz M., Wielckens K., Erdmann E., Schneider C. (2004). Use of NT-proBNP in routine testing and comparison to BNP. Eur. J. Heart Fail..

[7] Januzzi J.L., van Kimmenade R., Lainchbury J., Bayes-Genis A., Ordonez-Llanos J., Santalo-Bel M., Pinto Y.M., Richards M. (2006). NT-proBNP testing for diagnosis and short-term prognosis in acute destabilized heart failure: An international pooled analysis of 1256 patients: The International Collaborative of NT-proBNP Study. Eur. Heart J..

[8] Hall C. (2005). NT-ProBNP: The mechanism behind the marker. J. Card. Fail..

[9] Sahoo R.K., Singh S.K., Mane R.S., Varma S. (2023). Nanomaterials for Biosensing Applications in the Medical Field. Nanomaterials for Sustainable Development: Opportunities and Future Perspectives.

[10] Lee Y.-Y., Sriram B., Wang S.-F., Kogularasu S., Chang-Chien G.-P. (2023). A comprehensive review on emerging role of rare earth oxides in electrochemical biosensors. Microchem. J..

[11] Haidyrah A.S., Sundaresan P., Venkatesh K., Ramaraj S.K., Thirumalraj B. (2021). Fabrication of functionalized carbon nanofibers/carbon black composite for electrochemical investigation of antibacterial drug nitrofurantoin. Colloids Surf. A Physicochem. Eng. Asp..

[12] Thirumalraj B., Jaihindh D.P., Alaswad S.O., Sudhakaran M., Selvaganapathy M., Alfantazi A., Choe H., Kwon K. (2022). Fabricating BiOCl/BiVO4 nanosheets wrapped in a graphene oxide heterojunction composite for detection of an antihistamine in biological samples. Environ. Res..

[13] Sakthivel K., Lee Y.-Y., Sriram B., Wang S.-F., George M., Chang-Chien G.-P., Sheu J.-K. (2023). Unlocking Catalytic Potential: Exploring the Impact of Thermal Treatment on Enhanced Electrocatalysis of Nanomaterials. Angew. Chem..

[14] Sriram B., Kogularasu S., Wang S.-F., Chang-Chien G.-P. (2023). The Fabrication of a La2Sn2O7/f-HNT Composite for Non-Enzymatic Electrochemical Detection of 3-Nitro-l-tyrosine in Biological Samples. Biosensors.

[15] Akilarasan M., Kogularasu S., Chen S.-M., Chen T.-W., Lou B.-S. (2018). A novel approach to iron oxide separation from e-waste and bisphenol A detection in thermal paper receipts using recovered nanocomposites. RSC Adv..

[16] Alawieh H., El Chemaly T., Alam S., Khraiche M. (2019). Towards point-of-care heart failure diagnostic platforms: BNP and NT-proBNP biosensors. Sensors.

[17] Liu H.-L., Tseng Y.-T., Lai M.-C., Chau L.-K. (2022). Ultrasensitive and rapid detection of N-terminal pro-B-type natriuretic peptide (NT-proBNP) using fiber optic nanogold-linked immunosorbent assay. Biosensors.

[18] Pollok N.E., Rabin C., Walgama C.T., Smith L., Richards I., Crooks R.M. (2020). Electrochemical detection of NT-proBNP using a metalloimmunoassay on a paper electrode platform. ACS Sens..

[19] Wilkins M.D., Turner B.L., Rivera K.R., Menegatti S., Daniele M. (2018). Quantum dot enabled lateral flow immunoassay for detection of cardiac biomarker NT-proBNP. Sens. Bio-Sens. Res..

[20] Tang C., Zhang J.-X., Chen D.-N., He J.-W., Wang A.-J., Feng J.-J. (2022). Ultrasensitive label-free electrochemical immunosensor of NT-proBNP biomarker based on branched AuPd nanocrystals/N-doped honeycombed porous carbon. Bioelectrochemistry.

[21] Jensen J., Ma L.-P., Fu M.L., Svaninger D., Lundberg P.-A., Hammarsten O. (2010). Inflammation increases NT-proBNP and the NT-proBNP/BNP ratio. Clin. Res. Cardiol..

[22] Seino Y., Ogawa A., Yamashita T., Fukushima M., Ogata K.i., Fukumoto H., Takano T. (2004). Application of NT-proBNP and BNP measurements in cardiac care: A more discerning marker for the detection and evaluation of heart failure. Eur. J. Heart Fail..

[23] McDonagh T., Holmer S., Raymond I., Luchner A., Hildebrant P., Dargie H. (2004). NT-proBNP and the diagnosis of heart failure: A pooled analysis of three European epidemiological studies. Eur. J. Heart Fail..

[24] Palazzuoli A., Gallotta M., Quatrini I., Nuti R. (2010). Natriuretic peptides (BNP and NT-proBNP): Measurement and relevance in heart failure. Vasc. Health Risk Manag..

[25] Vergaro G., Gentile F., Aimo A., Januzzi Jr J.L., Richards A.M., Lam C.S., de Boer R.A., Meems L.M., Latini R., Staszewsky L. (2022). Circulating levels and prognostic cut-offs of sST2, hs-cTnT, and NT-proBNP in women vs. men with chronic heart failure. ESC Heart Fail..

[26] Biasucci L.M., Maino A., Grimaldi M.C., Cappannoli L., Aspromonte N. (2021). Novel biomarkers in heart failure: New insight in pathophysiology and clinical perspective. J. Clin. Med..

[27] John R.V., Devasiya T., VR N., Adigal S., Lukose J., Kartha V., Chidangil S. (2022). Cardiovascular biomarkers in body fluids: Progress and prospects in optical sensors. Biophys. Rev..

[28] Wu L., Shao P., Gao Z., Zhang S., Ma J., Bai J., Wei Y. (2023). Homocysteine and Lp-PLA2 levels: Diagnostic value in coronary heart disease. Medicine.

[29] Hua M., Chen W.-Y., Wang L.-H., Zou X.-H., Mao L.-L. (2023). The value of serum Lp-PLA2 combined with MPO in the diagnosis of cerebral infarction caused by large artery atherosclerosis. Clin. Neurol. Neurosurg..

[30] Dada N., Kim N.W., Wolfert R.L. (2002). Lp-PLA2: An emerging biomarker of coronary heart disease. Expert Rev. Mol. Diagn..

[31] Welsh P., Campbell R.T., Mooney L., Kimenai D.M., Hayward C., Campbell A., Porteous D., Mills N.L., Lang N.N., Petrie M.C. (2022). Reference ranges for NT-proBNP (N-terminal pro-B-type natriuretic peptide) and risk factors for higher NT-proBNP concentrations in a large general population cohort. Circ. Heart Fail..

[32] Halima H.B., Bellagambi F.G., Hangouët M., Alcacer A., Pfeiffer N., Heuberger A., Zine N., Bausells J., Elaissari A., Errachid A. (2023). A novel electrochemical strategy for NT-proBNP detection using IMFET for monitoring heart failure by saliva analysis. Talanta.

[33] Goryacheva O.A., Ponomaryova T.D., Drozd D.D., Kokorina A.A., Rusanova T.Y., Mishra P.K., Goryacheva I.Y. (2022). Heart failure biomarkers BNP and NT-proBNP detection using optical labels. TrAC Trends Anal. Chem..

[34] Myhre P.L., Vaduganathan M., Claggett B.L., Miao Z.M., Jhund P.S., de Boer R.A., Hernandez A.F., Inzucchi S.E., Kosiborod M.N., Lam C.S. (2022). Influence of NT-proBNP on efficacy of dapagliflozin in heart failure with mildly reduced or preserved ejection fraction. Heart Fail..

[35] Ouyang M., Tu D., Tong L., Sarwar M., Bhimaraj A., Li C., Cote G.L., Di Carlo D. (2021). A review of biosensor technologies for blood biomarkers toward monitoring cardiovascular diseases at the point-of-care. Biosens. Bioelectron..

[36] Qian Y., Feng J., Fan D., Zhang Y., Kuang X., Wang H., Wei Q., Ju H. (2019). A sandwich-type photoelectrochemical immunosensor for NT-pro BNP detection based on F-Bi_2_WO_6_/Ag_2_S and GO/PDA for signal amplification. Biosens. Bioelectron..

[37] Wang C., Zhu W., Yan T., Yang L., Kuang X., Du B., Pang X., Wei Q. (2018). Novel electrochemiluminescent platform based on gold nanoparticles functionalized Ti doped BiOBr for ultrasensitive immunosensing of NT-proBNP. Sens. Actuators B Chem..

[38] Nekrasov N., Kudriavtseva A., Orlov A.V., Gadjanski I., Nikitin P.I., Bobrinetskiy I., Knežević N.Ž. (2022). One-Step Photochemical Immobilization of Aptamer on Graphene for Label-Free Detection of NT-proBNP. Biosensors.

[39] Shi L., Li X., Zhu W., Wang Y., Du B., Cao W., Wei Q., Pang X. (2017). Sandwich-type electrochemiluminescence sensor for detection of NT-proBNP by using high efficiency quench strategy of Fe_3_O_4_@PDA toward Ru(bpy)_3_^2+^ coordinated with silver oxalate. ACS Sens..

[40] Harpaz D., Koh B., Seet R.C., Abdulhalim I., Tok A.I. (2020). Functionalized silicon dioxide self-referenced plasmonic chip as point-of-care biosensor for stroke biomarkers NT-proBNP and S100β. Talanta.

[41] Mao L., Yuan R., Chai Y., Zhuo Y., Xiang Y. (2011). Signal-enhancer molecules encapsulated liposome as a valuable sensing and amplification platform combining the aptasensor for ultrasensitive ECL immunoassay. Biosens. Bioelectron..

[42] Zhuo Y., Yi W.-J., Lian W.-B., Yuan R., Chai Y.-Q., Chen A., Hu C.-M. (2011). Ultrasensitive electrochemical strategy for NT-proBNP detection with gold nanochains and horseradish peroxidase complex amplification. Biosens. Bioelectron..

[43] Jiang C., Lai X., Han F., Gao Z., Yang H., Zhao X., Pang H., Qiao B., Pei H., Wu Q. (2023). Shape dependency of gold nanorods through TMB^2+^-mediated etching for the visual detection of NT-proBNP. RSC Adv..

[44] Vairaperumal T., Huang C.-C., Liu P.-Y. (2023). Optical Nanobiosensor-Based Point-of-Care Testing for Cardiovascular Disease Biomarkers. ACS Appl. Bio Mater..

[45] Harpaz D., Koh B., Marks R.S., Seet R.C., Abdulhalim I., Tok A.I. (2019). Point-of-Care surface plasmon resonance biosensor for stroke biomarkers NT-proBNP and S100β using a functionalized gold chip with specific antibody. Sensors.

[46] Liu Y., Wang H., Xiong C., Chai Y., Yuan R. (2017). An ultrasensitive electrochemiluminescence immunosensor for NT-proBNP based on self-catalyzed luminescence emitter coupled with PdCu@ carbon nanohorn hybrid. Biosens. Bioelectron..

[47] Li Y., Wang Y., Zhang N., Fan D., Liu L., Yan T., Yang X., Ding C., Wei Q., Ju H. (2019). Magnetic electrode-based electrochemical immunosensor using amorphous bimetallic sulfides of CoSnS_x_ as signal amplifier for the NTpro BNP detection. Biosens. Bioelectron..

[48] Li X., Lu P., Wu B., Wang Y., Wang H., Du B., Pang X., Wei Q. (2018). Electrochemiluminescence quenching of luminol by CuS in situ grown on reduced graphene oxide for detection of N-terminal pro-brain natriuretic peptide. Biosens. Bioelectron..

[49] Guo Q., Ding L., Li Y., Xiong S., Fang H., Li X., Nie L., Xiong Y., Huang X. (2022). Covalent organic framework-gold nanoparticle heterostructures amplified dynamic light scattering immunosensor for ultrasensitive detection of NT-proBNP in whole blood. Sens. Actuators B Chem..

[50] Cardoso A.G., Ahmed S.R., Keshavarz-Motamed Z., Srinivasan S., Rajabzadeh A.R. (2023). Recent advancements of nanomodified electrodes-towards Point-of-Care detection of cardiac biomarkers. Bioelectrochemistry.

[51] António M., Vitorino R., Daniel-da-Silva A.L. (2023). LSPR-Based Aptasensor for Rapid Urinary Detection of NT-proBNP. Biosensors.

[52] Beck F., Horn C., Baeumner A.J. (2022). Dry-reagent microfluidic biosensor for simple detection of NT-proBNP via Ag nanoparticles. Anal. Chim. Acta.

[53] Yang D., Ma J., Xue C., Wang L., Wang X. (2018). One-pot synthesis of poly(acrylic acid)-stabilized Fe_3_O_4_ nanocrystal clusters for the simultaneously qualitative and quantitative detection of biomarkers in lateral flow immunoassay. J. Pharm. Biomed. Anal..

[54] Peng Y., Raj N., Strasser J.W., Crooks R.M. (2022). Paper biosensor for the detection of NT-proBNP using silver nanodisks as electrochemical labels. Nanomaterials.

[55] Luo Q., Qian X., Mi X., Tu Y. (2022). A novel electrochemiluminescent immunosensor for the detection of NT-proBNP based on a Au/ZIF-67 nanocomposite. J. Electroanal. Chem..

[56] Goryacheva O.A., Kokorina A.A., Podkolodnaya Y.A., Mishra P.K., Goryacheva I.Y. (2023). Express test for NT-proBNP competitive detection based on lateral flow immunoassay using silanized fluorescent quantum dots. Talanta Open.

[57] Liang W., Li Y., Zhang B., Zhang Z., Chen A., Qi D., Yi W., Hu C. (2012). A novel microfluidic immunoassay system based on electrochemical immunosensors: An application for the detection of NT-proBNP in whole blood. Biosens. Bioelectron..

[58] Tai T.-Y., Sinha A., Sarangadharan I., Pulikkathodi A.K., Wang S.-L., Lee G.-Y., Chyi J.-I., Shiesh S.-C., Lee G.-B., Wang Y.-L. (2019). Design and demonstration of tunable amplified sensitivity of AlGaN/GaN high electron mobility transistor (HEMT)-based biosensors in human serum. Anal. Chem..

[59] He Y., Wang Y., Yang X., Xie S., Yuan R., Chai Y. (2016). Metal organic frameworks combining CoFe_2_O_4_ magnetic nanoparticles as highly efficient SERS sensing platform for ultrasensitive detection of N-terminal pro-brain natriuretic peptide. ACS Appl. Mater. Interfaces.

[60] Lin H.-H., Wang I.-S., Yen P.-W., Cheng H., Tsai H.-H., Liao H.-H., Lu S.-J., Chou F.-C., Lin C.-T. (2014). A CMOS Based Polysilicon Nanowire Biosensor Platform for Different Biological Targets. Procedia Eng..

[61] Munief W.-M., Lu X., Teucke T., Wilhelm J., Britz A., Hempel F., Lanche R., Schwartz M., Law J.K.Y., Grandthyll S. (2019). Reduced graphene oxide biosensor platform for the detection of NT-proBNP biomarker in its clinical range. Biosens. Bioelectron..

[62] Sinawang P.D., Harpaz D., Fajs L., Seet R.C.S., Tok A.I.Y., Marks R.S. (2017). Electrochemical impedimetric detection of stroke biomarker NT-proBNP using disposable screen-printed gold electrodes. EuroBiotech J..

[63] Liang W., Zhuo Y., Xiong C., Zheng Y., Chai Y., Yuan R. (2017). A sensitive immunosensor via in situ enzymatically generating efficient quencher for electrochemiluminescence of iridium complexes doped SiO_2_ nanoparticles. Biosens. Bioelectron..

[64] Zhao Y., Li L., Hu L., Zhang Y., Wu D., Ma H., Wei Q. (2019). An electrochemiluminescence immunosensor for the N-terminal brain natriuretic peptide based on the high quenching ability of polydopamine. Microchim. Acta.

[65] Zhang Y., Xu R., Kang Q., Zhang Y., Wei Q., Wang Y., Ju H. (2018). Ultrasensitive photoelectrochemical biosensing platform for detecting n-terminal pro-brain natriuretic peptide based on SnO_2_/SnS_2_/mpg-C_3_N_4_ amplified by PbS/SiO_2_. ACS Appl. Mater. Interfaces.

[66] Feng J., Li F., Li X., Wang Y., Fan D., Du B., Li Y., Wei Q. (2018). Label-free photoelectrochemical immunosensor for NT-proBNP detection based on La-CdS/3D ZnIn_2_S_4_/Au@ ZnO sensitization structure. Biosens. Bioelectron..

[67] Sinha A., Gopinathan P., Chung Y.-D., Shiesh S.-C., Lee G.-B. (2019). Simultaneous detection of multiple NT-proBNP clinical samples utilizing an aptamer-based sandwich assay on an integrated microfluidic system. Lab Chip.

[68] Halima H.B., Bellagambi F.G., Brunon F., Alcacer A., Pfeiffer N., Heuberger A., Hangouët M., Zine N., Bausells J., Errachid A. (2023). Immuno field-effect transistor (ImmunoFET) for detection of salivary cortisol using potentiometric and impedance spectroscopy for monitoring heart failure. Talanta.

[69] Tian J., Lu X., Cheng Y., Fang Z., Ren G., Liu N. (2021). Construction of high sensitivity electrochemiluminescence sensor and its application in nt-probnp detection. Mater. Express.

[70] Assunção A.S., Vidal M., Loyez M., Caucheteur C., Costa F.M., Mesquita-Bastos J., Pereira S.O., Leitão C. Unclad optical fiber tips for plasmonic biosensing of heart failure biomarker. Proceedings of the European Workshop on Optical Fibre Sensors (EWOFS 2023).

[71] Pu Q., Yang X., Guo Y., Dai T., Yang T., Ou X., Li J., Sheng S., Xie G. (2019). Simultaneous colorimetric determination of acute myocardial infarction biomarkers by integrating self-assembled 3D gold nanovesicles into a multiple immunosorbent assay. Microchim. Acta.

[72] Raj N., Crooks R.M. (2022). Plastic-based lateral flow immunoassay device for electrochemical detection of NT-proBNP. Analyst.

